# Quantifying the association between stroke and dementia: a bibliometric study

**DOI:** 10.3389/fneur.2024.1438699

**Published:** 2024-10-08

**Authors:** Xinyi Bian, Zibin Zhao, Xiaoping Gao

**Affiliations:** ^1^Department of Rehabilitation, The First Affiliated Hospital of Anhui Medical University, Hefei, China; ^2^The First School of Clinical Medicine, Bengbu Medical University, Bengbu, China

**Keywords:** stroke, dementia, bibliometric, CiteSpace, VOSviewer

## Abstract

**Background:**

Stroke and dementia are two serious neurological disorders in modern medicine. Studies have revealed a significant link between the two, but there is still a lack of bibliometric analysis in this area. The objective of this study is to use bibliometric analysis to investigate the connection between stroke and dementia, as well as to assess the current state of research in this field and identify future trends.

**Methods:**

The publications from the Web of Science were Collection and retrieved for the last 22 years (2002–2023). CiteSpace, VOSviewer, and the R package Bibliometrix were used to conduct bibliometric analysis. GraphPad Prism was used to plot.

**Results:**

A total of 1,309 publications were included in the analysis. The number of articles on dementia and stroke has continued to grow steadily over the past 22 years. While China is the country with the most articles, the most influential and widely researched countries are England and the United States. The keyword analysis illustrates that the prevention of dementia through stroke prevention is a major focus and trend in this research area.

**Conclusion:**

This study provides a visual analysis method for measuring the association between stroke and dementia, and examines the current state of research in this area and future research trends. In the future, dementia caused by stroke needs to be emphasized, and prevention of dementia through stroke prevention is a research priority.

## 1 Introduction

Stroke refers to a disease in which there is an acute disruption of blood flow to the brain, leading to insufficient or interrupted blood supply and subsequent damage to brain tissue. Approximately 70% of strokes are caused by blockage of the major cerebral arteries, with occlusion of large arteries often resulting from thrombosis or atrial fibrillation and occlusion of a small artery due to small vessel disease. Stroke is currently the second most common cause of death globally and stands as the primary origin of long-term disability (1–4). Due to the increasing prevalence of risk factors such as hypertension, obesity, hyperlipidemia, smoking, and drug abuse, the incidence of stroke among young people has been increasing rapidly (5, 6). The trend of strokes affecting younger individuals is becoming increasingly concerning. The main symptoms of a stroke include the sudden onset of weakness or numbness in the face, arms, or legs, difficulty in speaking or understanding, loss or blurring of vision, and severe headache.

Dementia is a progressive neurodegenerative disorder primarily affects memory, cognitive function, and behavior. It is characterized by symptoms such as memory loss, language difficulties, decreased spatial orientation, and impaired judgment and abstract thinking. Dementia can be caused by various factors, including Alzheimer's disease, cognitive impairment, and other underlying condition (7, 8). Unfortunately, there is currently no cure for dementia, but medications and non-medication treatments are available that can help manage symptoms and improve the quality of life for patients. The impact of dementia is profound, not only for the individuals affected but also for their families and society at large (9, 10).

According to the Global Burden of Disease report, in 2019, neurological disorders continued to be the predominant cause of Disability Adjusted Life Years (DALYs), constituting 10.8% of the total burden of DALYs attributable to all causes. Stroke was responsible for 69.8% of deaths due to neurological disorders and accounted for 52.3% of the neurological DALYs (11). Of these, stroke and dementia dominate the list of neurological diseases (1). Due to the same risk factors for stroke and dementia, there is a high prevalence of cognitive impairment and dementia symptoms after a stroke (12–16). According to studies, about 30–50% of stroke patients will develop cognitive impairment and dementia symptoms after a stroke (17, 18). People who develop dementia immediately after stroke have early-onset dementia (13, 19), but those who do not initially have dementia are also at risk for delayed-onset dementia in the long term, with an approximately 1-to-8-fold increased dementia risk ranging from 3 to 16 years after stroke (20–24). A study of stroke prevention showed that population-wide prevention strategies aim to reduce the incidence of stroke by reducing the average level of exposure to disease-causing risk factors. If implemented effectively, these strategies can prevent up to 50–90% of all strokes within 5 years. It is estimated that approximately 40% of dementia cases could be prevented by targeting modifiable, primarily cardiovascular risk factors (24, 25). Since stroke and dementia often occur together and are at risk for each other, preventing stroke can also prevent certain dementias (26–28).

There is a lack of objective and comprehensive reporting through studies of publication trends, keyword hotspots, and common collaborative networks. The purpose of this paper is to analyze the relationship between stroke and dementia from 2002 to 2022 using a bibliometric analysis system. Bibliometric analysis is a powerful approach that combines mathematical and statistical methods with data visualization to analyze various aspects of scholarly publications. This analysis can provide insights into annual publication trends, countries or regions involved, institutions, journals, authors, and co-citations (29).

## 2 Materials and methods

### 2.1 Search strategy

**Figure 1:**
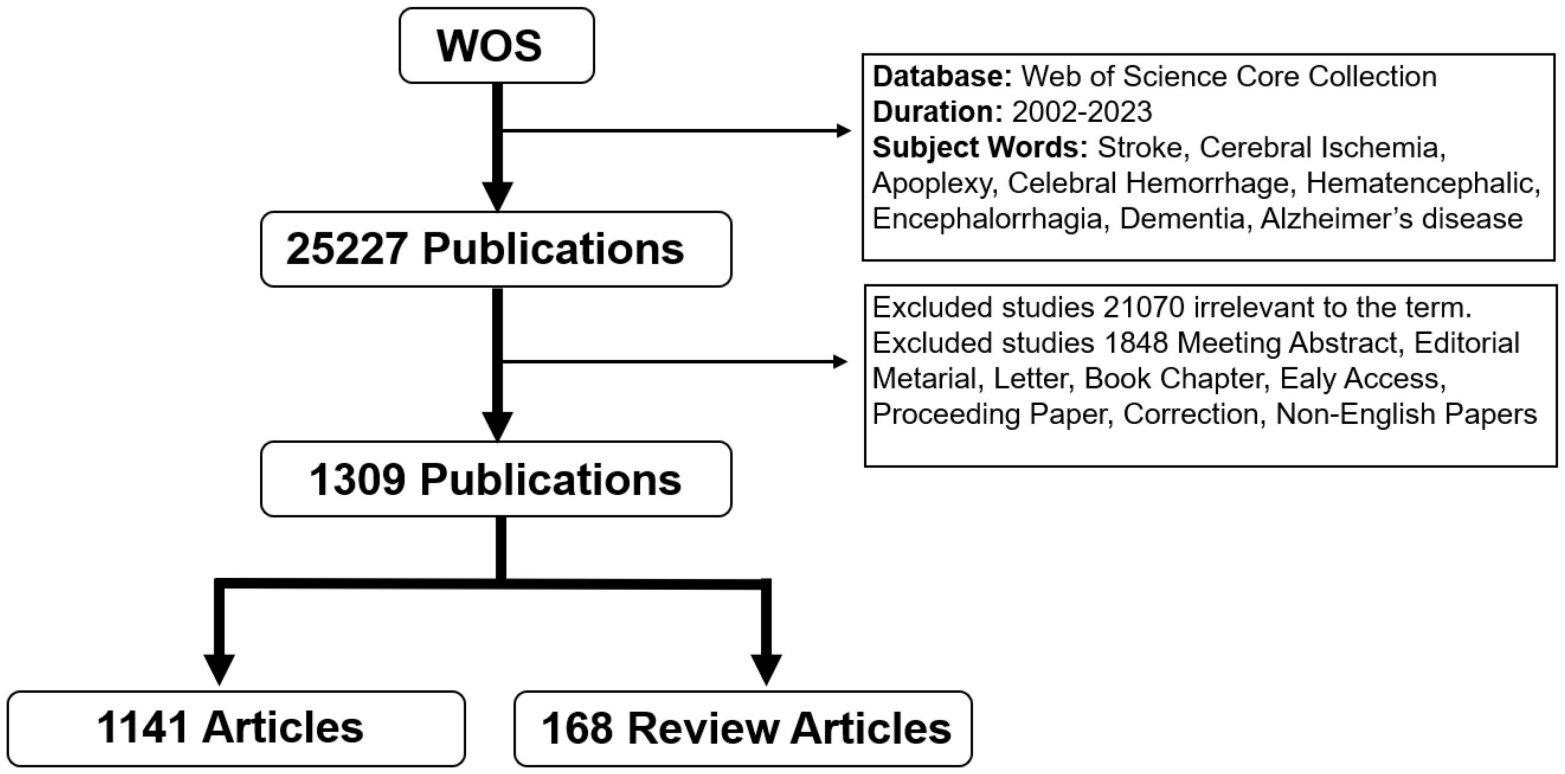
Flowchart outlining the screening and analysis process.

Data were gathered for this study from the Web of Science Core Collection (WoSCC) between 2002 and 2023. The search was conducted on 1 April 2024. The search terms are listed below: [TS = (stroke) OR TS = (cerebral Ischemia) OR TS = (apoplexy) OR TS = (cerebrovascular accident) OR TS = (cerebral hemorrhage) OR TS = (Hematencephalic) OR TS= (encephalorrhagia)] AND [TS= (dementia) OR TS = (Alzheimer's disease) OR TS = (cognitive impairment)]. Filter out irrelevant articles by reading the title and abstract. English-language articles and review articles were then analyzed in various forms of relevant publications (meeting abstracts, editorial material, letters, book chapters, earlier access, proceeding papers, corrections, and non-English papers). In total, 1,141 articles and 168 review articles were accessed and studied. The retrieval strategy is shown in Figure 1.

### 2.2 Bibliometric analysis and visualization

Data plotting and statistical analysis were performed using GraphPad Prism 9. VOSviewer (1.6.19) and CitaSpace (6.2.R3) software were selected to construct and visualize this study's bibliometric network of publications. VOSviewer provides a detailed analysis of bibliographic coupling, co-citation, and co-occurrence analysis. CiteSpace was developed by Professor Chen C. Citespace is used to construct double-graph overlays of journals, cluster analysis of co-cited keywords, and detection of citation outbreak sharp keywords. In addition, we chose the R package “bibliometrix” to visualize collaborative networks of highly productive authors, visualize publication production between countries, and map international collaborations between countries. The H-index is an indicator of a scholar's or country's scientific impact reflecting the fact that a scholar or country has published H publications and each publication has been cited at least H times by other publications (30).

## 3 Results

### 3.1 Analysis of publication output

**Figure 2:**
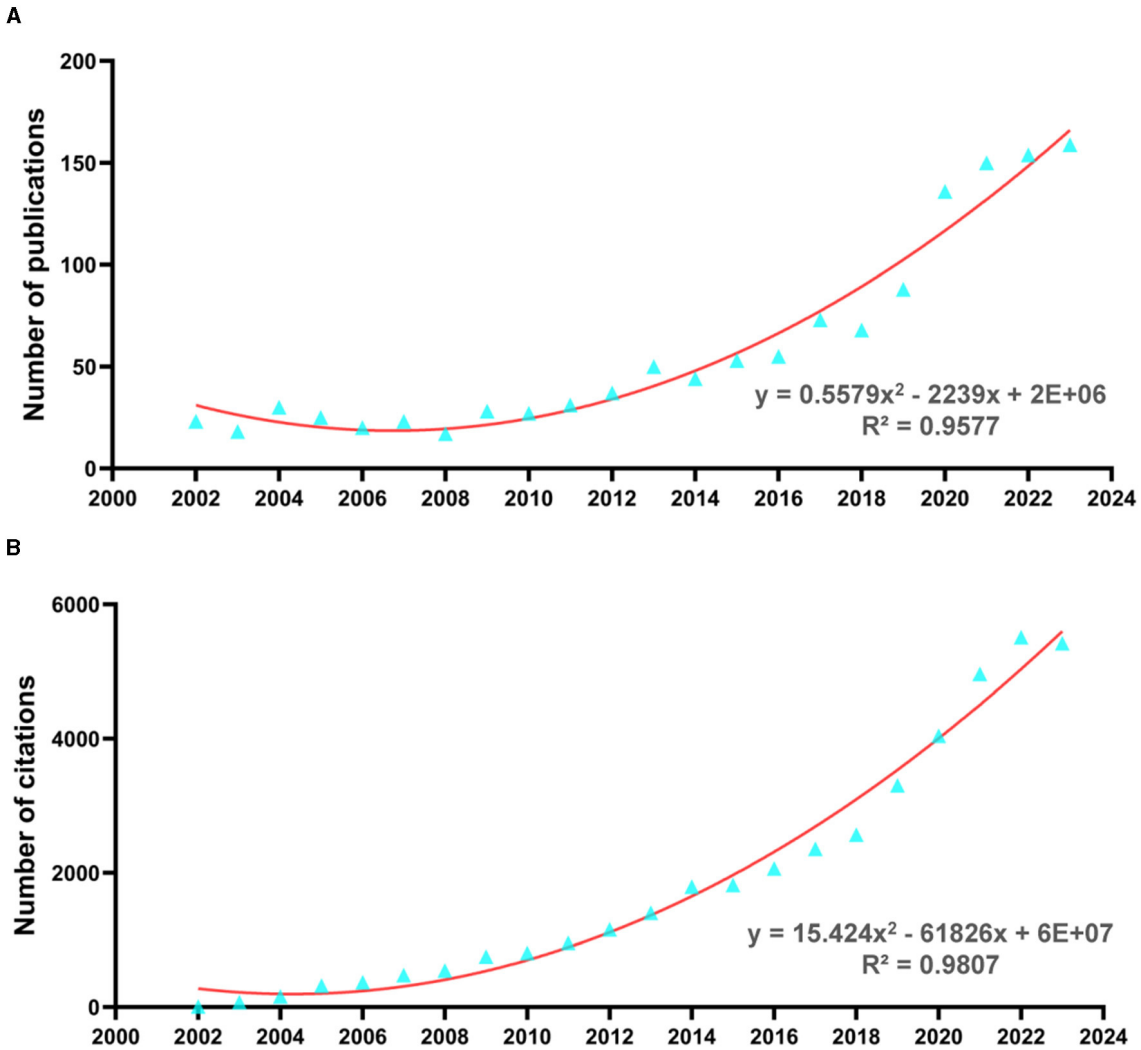
Number of publications and citations. **(A)** The annual number of publications on stroke and dementia research from 2002 to 2023. **(B)** The annual number of citations for research on stroke and dementia from 2002 to 2023.

Based on the search criteria, 1,309 articles related to the association between stroke and dementia were screened. Figure 2A shows the number of articles in this field was stable from 2002 to 2012 and has increased rapidly since 2012. The distribution of annual publications related to stroke and dementia from 2002 to 2023. The positive incremental increase in annual publications and significant correlation in time trend of publications (R^2^ = 0.9577, *p* < 0.0001), implies faster growth of publication reviews in the future. Figure 2B shows the annual citation distribution of the included studies, with the number of citations increasing from 8 in 2002 to 5,418 in 2023. There was an overall positive incremental increase in the number of annual citations, with a significant correlation in the time trend of citations (R^2^ = 0.9807, *p* < 0.0001).

### 3.2 Analysis of countries and international cooperation

**Figure 3:**
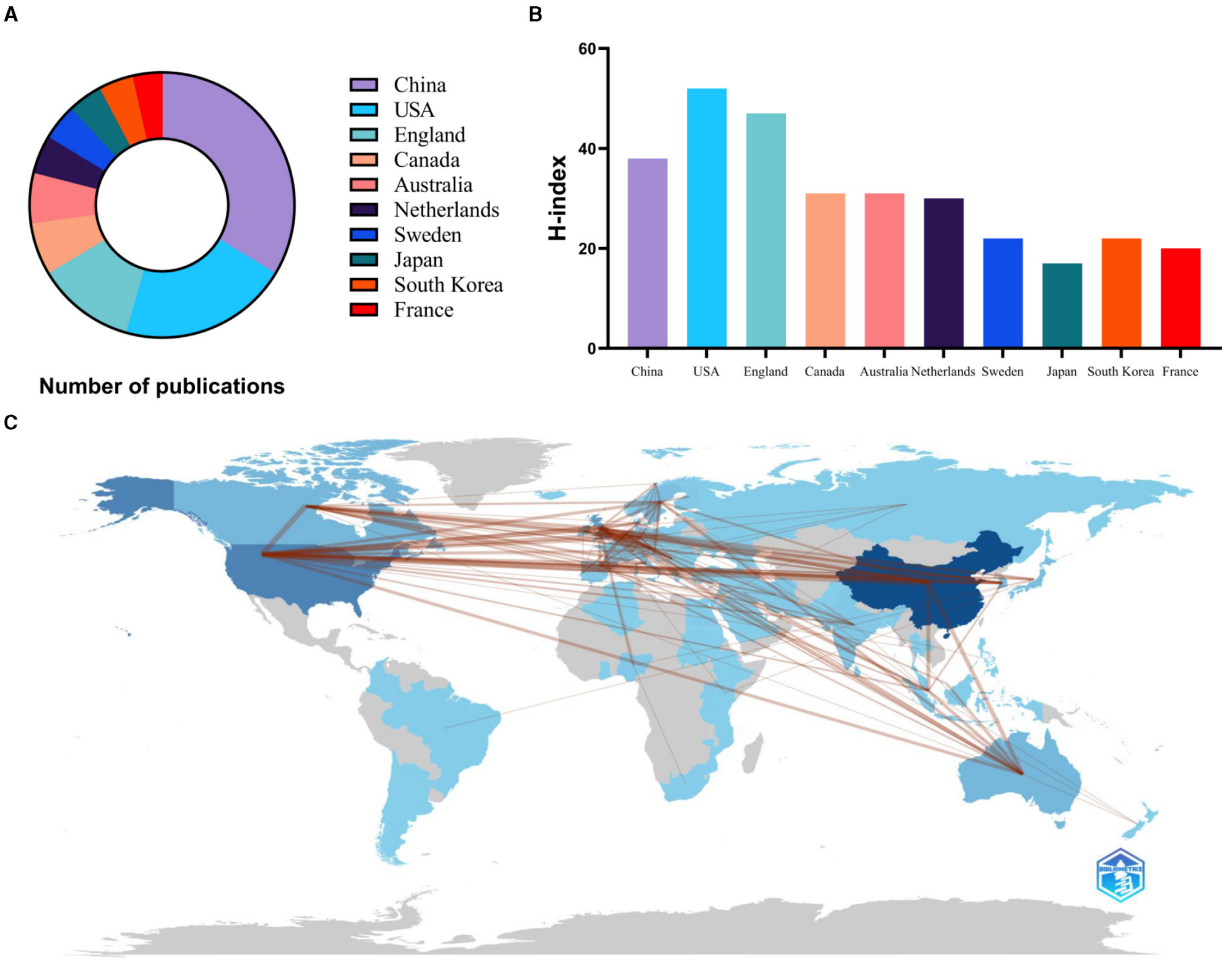
Top 10 countries of publications and national cooperation networks in terms of volume. **(A)** The hollow pie chart illustrates the percentage of publications from the top 10 countries in terms of volume of publications. **(B)** H-index for the top 10 countries in terms of publications. **(C)** Country cooperation network map.

From 2002 to 2023, 80 countries are involved in research in this field. The top ten countries in terms of publications are shown in Figure 3A, with China having the highest number of publications (423), followed by the United States (261) and England (151). This indicates that these countries have fruitful research results and strong research strength in this field.

**Figure 4:**
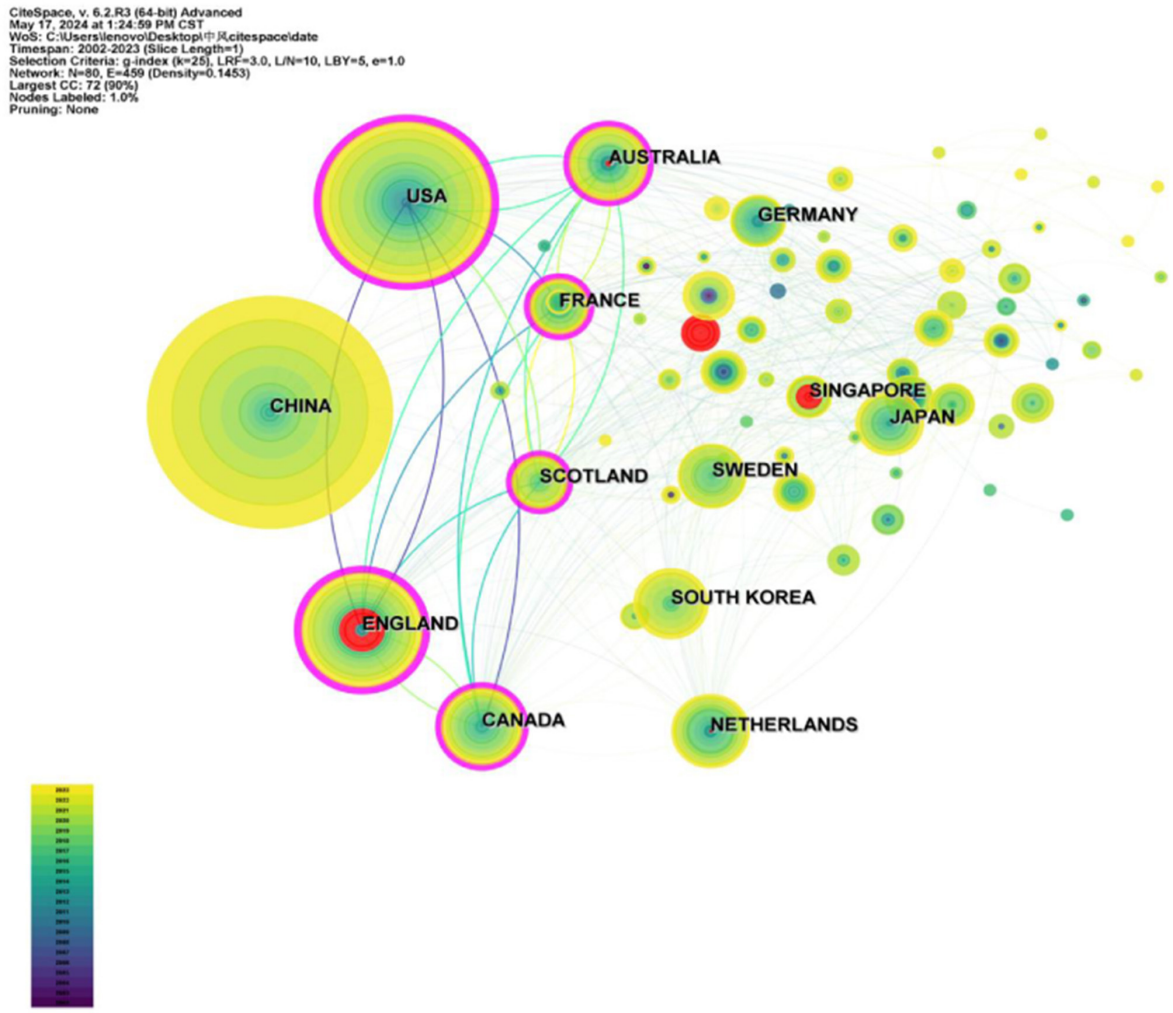
National co-authorship networks.

The H-index of the United States is ranked first (Figure 3B). Although China ranked first in terms of the number of publications, its H-index was 38, indicating limited academic impact and the need for continued efforts to improve the quality of research. The country cooperation map shows that cooperation mainly exists in China and the US (Figure 3C). According to Figure 4, collaboration between countries or regions in this area is generally close, with a network density of 0.1453. Network density is a metric used to quantify the extent of connectivity between nodes in a network. It is defined as the ratio of actual edges to possible edges in a network. In general, network density values vary from 0 to 1. In this study, the network density is 0.1453, indicating that the network nodes have some degree of connectivity with each other. However, there is potential for enhancement. Many countries or regions tend to cooperate and exchange with each other.

### 3.3 Analysis of institution

Between 2002 and 2023, 435 institutions conducted research in the area of stroke and dementia. Among them, the Capital Medical University is the most efficient institution in terms of the number of articles published, with strong centrality, indicating that the Capital Medical University have a wide range of research and strong influence on the name (Table 1). Three of the top five national institutions in terms of publications are located in England. In terms of the starting year of publication, institutions from Sweden and the UK were the earliest, beginning in 2002. While China's Capital Medical University was the latest to start publishing in 2013. Nevertheless, it is very active in researching stroke and dementia and has the potential to become a leader in the field. Notably, Harvard maintains strong ties with other institutions.

**Table 1:** Top 10 institutions for publications.

**Rank**	**Institutions**	**Countries**	**Publications**	**H-index**	**Total cited**	**Year**
1	Capital Medical University	China	41	11	381	2013
2	University of Toronto	Canada	40	20	2,333	2005
3	University of Oxford	England	39	25	3,239	2009
4	Karolinska Institute	Sweden	38	17	1,129	2002
5	Newcastle University	England	37	21	2,717	2007
6	University of London	England	37	19	1,712	2002
7	Chinese University of Hong Kong	China	32	17	1,121	2004
8	Harvard University	USA	30	16	1,246	2010
9	Western University	Canada	28	14	774	2007
10	National University of Singapore	Singapore	27	18	1,325	2007

**Figure 5:**
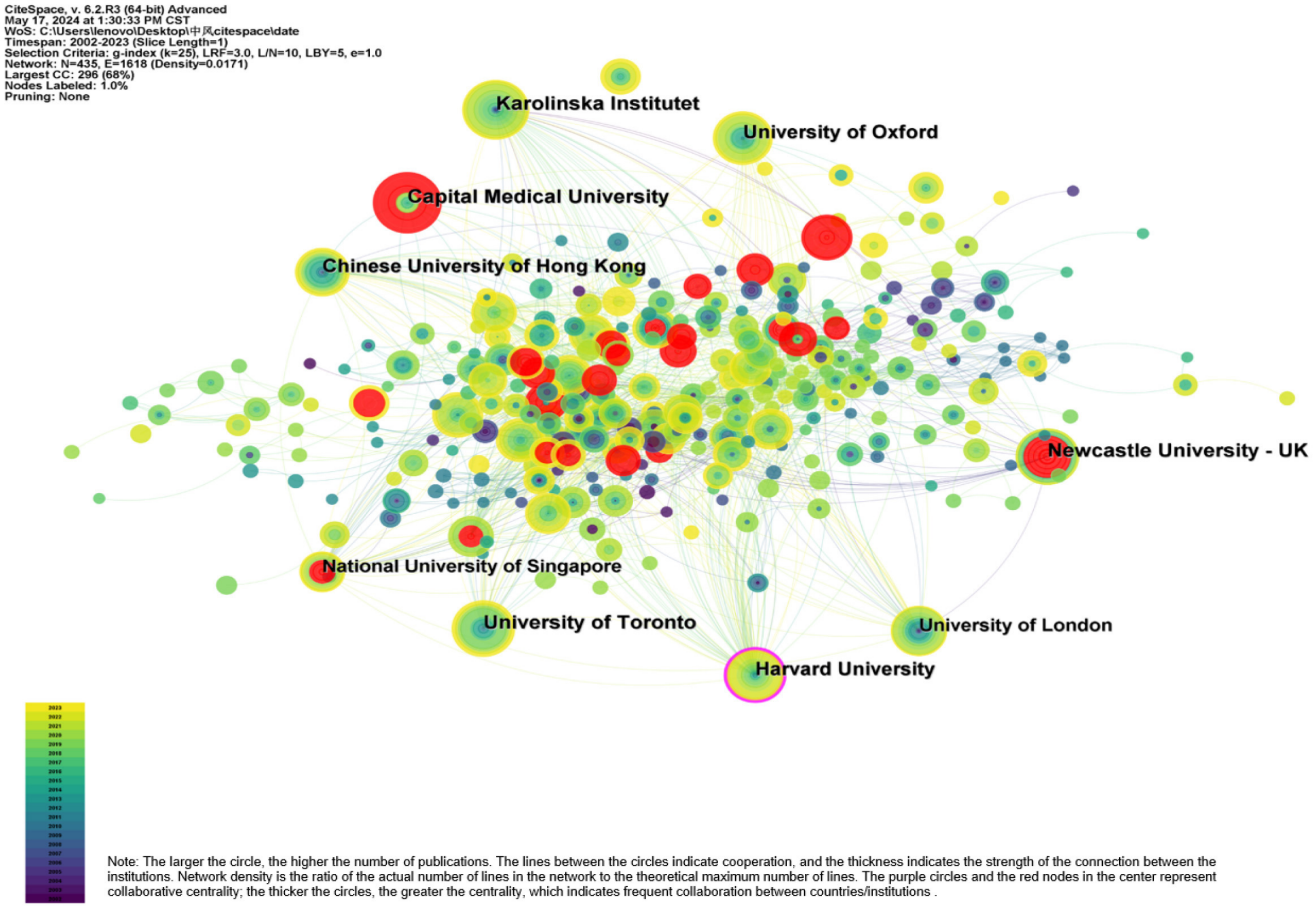
Institutional co-authorship networks.

To present the cooperation between countries/institutions, a network diagram of institutional cooperation was generated using CiteSpace, with the number of years set to 1 for each slice. The results are displayed in Figure 5. England has strong collaborative centrality, which indicates that it plays an important role in the knowledge transfer process in this area.

### 3.4 Analysis of journals and research areas

The number of publications published in a total of 443 reputed journals amounted to 1,309. The journal with the highest number of publications was Stroke (80 publications, IF: 8.4, JCR: Q1). While the Journal of Alzheimer's Disease (40 publications, IF:4, JCR: Q2) and the Journal of Stroke Cerebrovascular Disease (38 publications, IF = 2.5, JCR: Q3) were close behind. Stroke journals have the highest number of publications, H-index, and number of citations, while Journal of the Neurological Sciences and Neurology publications were cited just below stroke journals (Table 2).

**Table 2:** Top 10 journals for publications.

**Rank**	**Journals**	**Publications**	**Total cited**	**H-index**	**IF**	**JCR**	**Year**
1	Stroke	80	9,616	45	8.4	Q1	2002
2	Journal of Alzheimer's Disease	40	1,156	18	4	Q2	2007
3	Journal of Stroke Cerebrovascular Disease	38	415	11	2.5	Q3	2010
4	Frontiers in Neurology	36	372	11	3.4	Q2	2013
5	Neurology	31	1,584	23	10.1	Q1	2002
6	Journal of the Neurological Sciences	29	1,222	18	4.4	Q2	2002
7	International Journal of Stroke	28	755	14	6.7	Q1	2010
8	PLoS ONE	24	727	15	3.7	Q2	2010
9	Frontiers in Aging Neuroscience	22	224	9	4.8	Q2	2005
10	Journal of Neurosurgery and Psychiatry	18	1,005	16	11.1	Q1	2004

**Figure 6:**
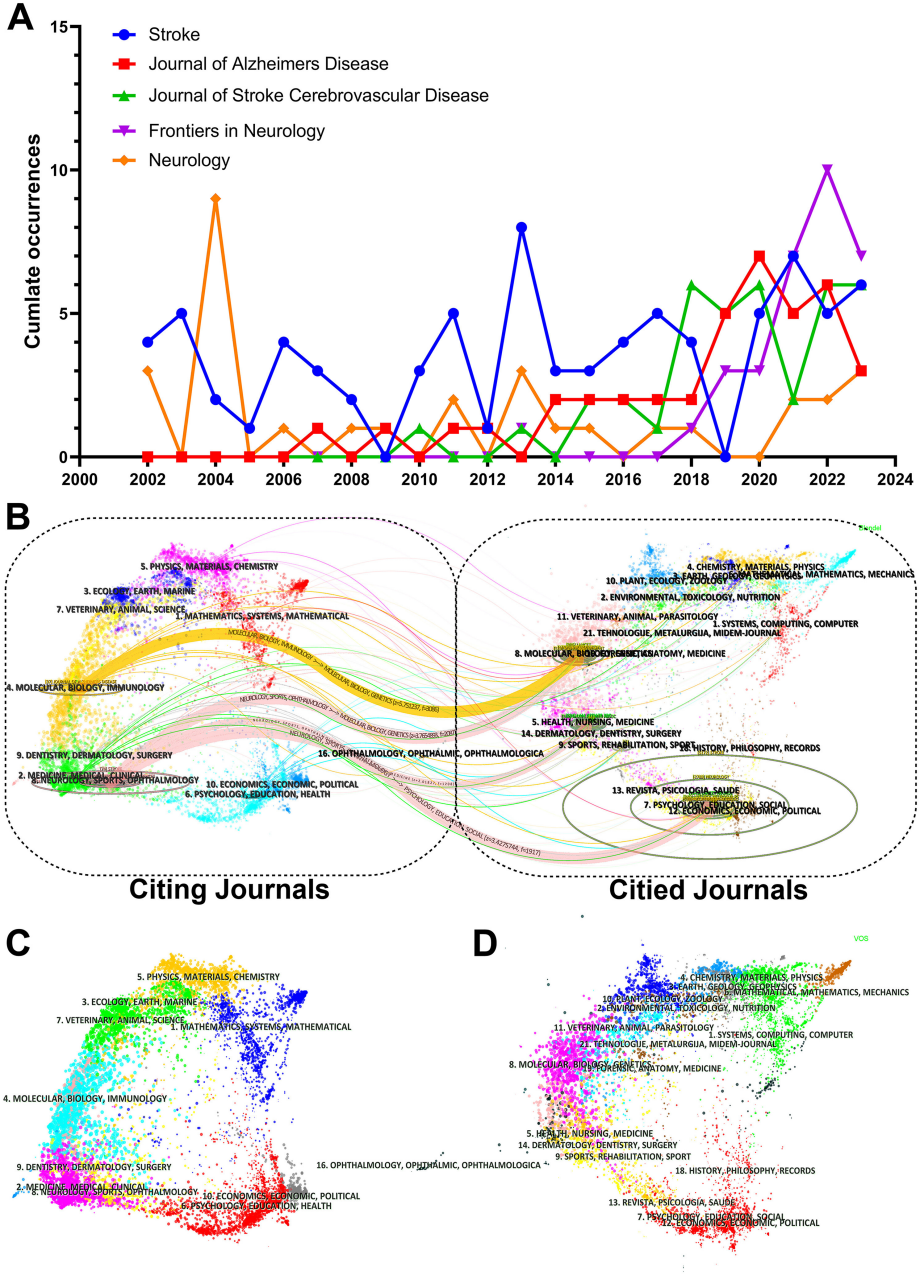
Total annual journal publications and journal overlay charts. **(A)** Top six journals in terms of number of publications Total annual publications from 2002 to 2023. **(B)** Double map overlay of journals. The set of cited journals is on the left and the set of cited journals is on the right, with colored paths indicating citation relationships. **(C)** Citing journals overlay. **(D)** Cited journals overlay.

The top five journals in terms of publications can be seen in Figure 6A, which shows that the top fix journals in terms of publications have continued to publish in the field steadily from year to year. Frontiers in Neurology has been quite active in the field, despite its late start in terms of publications on the field. The dual-map overlay atlas is a tool that visually links the cited journals on the left to the cited journals on the right, and also specifically reveals the citation relationships and subject distributions in the literature. The dual-map overlay atlas results show a relative concentration of journals studying the topic. The source journals for citations and references are mainly in the fields of Psychology/Education/Social, Health/Nursing/Medicine, and Molecular/Biology/Genetics, where the citation chains are generated. The areas of environment/toxicology/nutrition, dentistry/dermatology/surgery, and mathematics/systems/mathematical research are likely to become hotspots for research in the future (Figures 6B–D).

### 3.5 Analysis of authors

**Figure 7:**
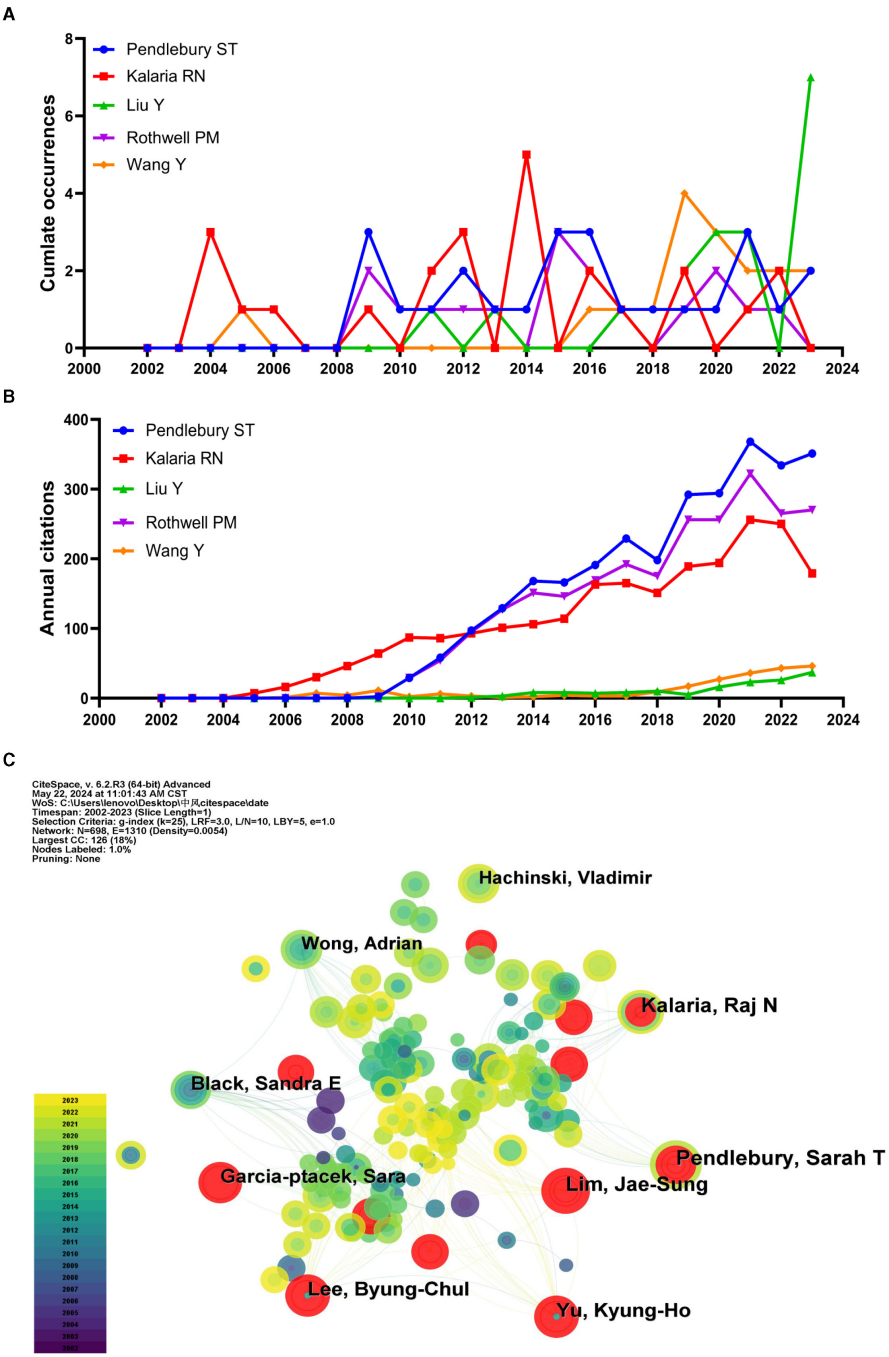
The authors' total annual publications, annual citations, and collaborative network diagrams. **(A)** Total number of annual publications by the top five authors in terms of number of publications. **(B)** Annual citations for the top five authors in terms of publications. **(C)** Author Co-occurrence Network.

A total of 6,061 authors contributed to these 1,309 articles. The top ten authors in terms of publications are shown in Table 3. Pendlebury ST ranked first with 25 publications, followed by Kalaria RN with 24 and Liu Y with 18. Rothwell PM, Wang Y, Wang YJ, and Wong A each published 17 articles. In terms of citations, Pendlebury ST's work has been cited 2,927 times, the highest among all authors, followed by Rothwell PM with 2,531 citations and Kalaria RN with 2,297 citations. This underscores the significant contributions of these authors to the field. When it comes to the annual publication of papers, the majority of the leading five authors in the field maintain a consistent output. It is important to mention that despite Pendlebury ST's tardiness in posting, this author is highly engaged in the field (Figure 7A). Ever since Pendlebury Sarah T began posting in 2009, her citations have experienced a significant increase (Figure 7B). In the co-authored map, some researchers and other active scholars are independently dispersed. Pendlebury ST and Kalaria Raj are central scholars, but they do not have access to all the study groups (Figure 7C). There is a lack of collaboration between center authors and other scholars in the field of stroke and dementia.

**Table 3:** Top 10 authors for publications.

**Rank**	**Authors**	**Publications**	**Total cited**	**H-index**	**Year**
1	Pendlebury ST	25	2,927	21	2009
2	Kalaria RN	24	2,297	16	2004
3	Liu Y	18	165	6	2011
4	Rothwell PM	17	2,531	14	2009
5	Wang Y	17	241	9	2005
6	Wang YJ	17	218	8	2011
7	Wong A	17	619	11	2005
8	Li J	16	296	10	2011
9	Hachinski V	15	1,597	11	2003
10	Lim JS	15	297	8	2017

### 3.6 The most frequently cited articles analysis

**Figure 8:**
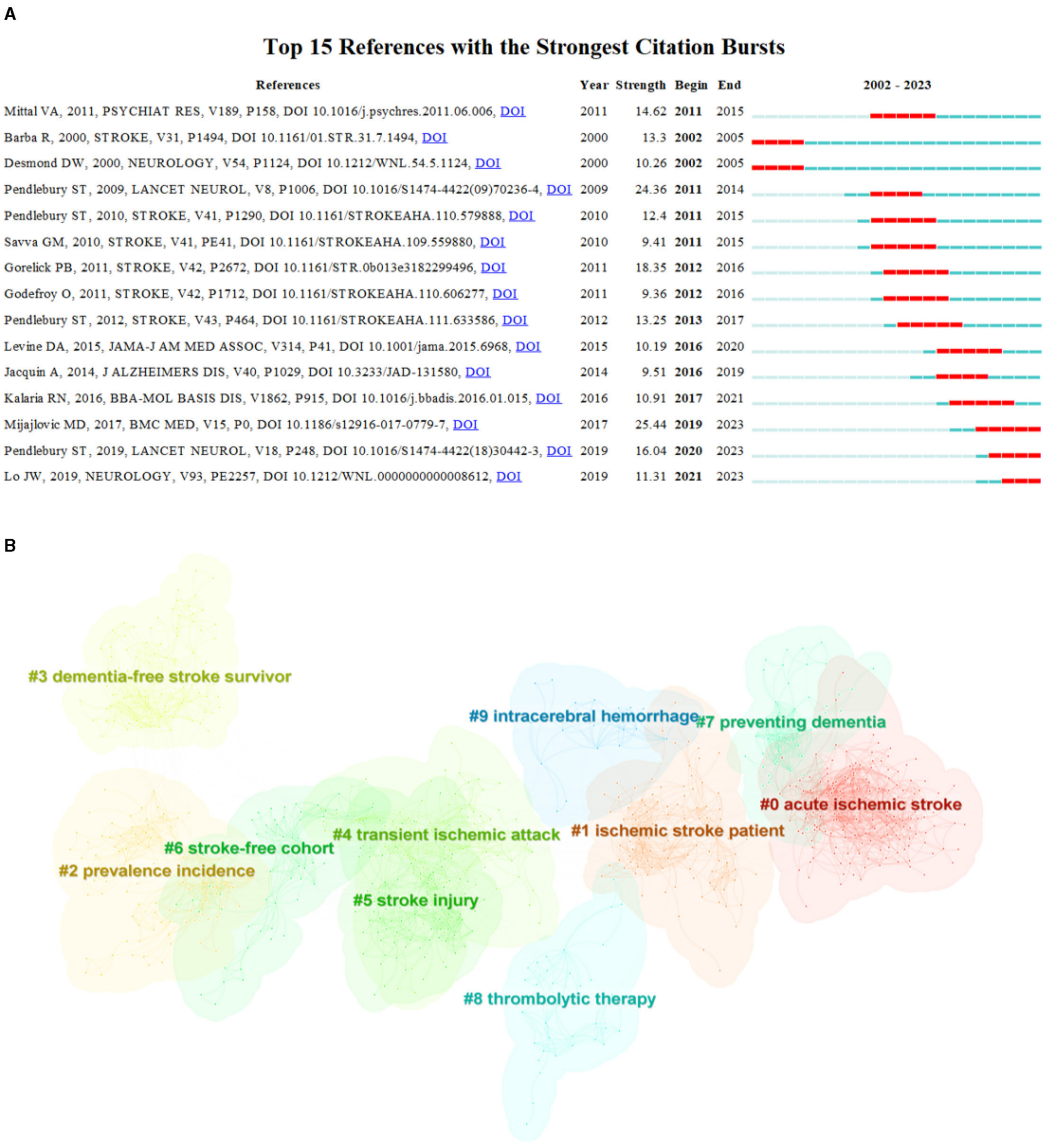
Top 15 References and Reference Clusters with the Strongest Citation Bursts in Stroke and Dementia Research. **(A)** Top 15 references with the most cited outbreaks. **(B)** Cluster results of references.

Table 4 presents the top ten co-cited references, each of which has been cited at least 316 times. Among them, three articles received over 1,000 citations. Upon analyzing these highly cited publications, it was found that they mainly dealt with the vascular factors contributing to stroke and dementia, prevalence, guideline, and criteria. Burst detection can be used to identify references that have received collective attention during a specific time period. In Figure 8A, it is shown the top 15 references that have the highest number of outbreaks. Among them, four articles authored by Pendlebury were widely cited from 2009 to 2023, which cover the incidence of post-stroke dementia, screening post-stroke dementia populations, and neuropsychology. Citation bursts is used to describe a sudden and significant increase in the frequency of citations to a particular piece of literature over a specific period of time. This phenomenon is often indicative of the widespread interest and importance of that piece of literature during the given period. With the help of CiteSpace, the co-cited references have been clustered to show the correlation between acute ischemic stroke, ischemic stroke patient, prevalence incidence, dementia-free stroke survivor, transient ischemic attack, stroke injury, stroke-free cohort, preventing dementia, thrombolytic therapy, thrombolytic therapy, and intracerebral hemorrhage (Figure 8B).The Cluster Results method groups literature based on similarities in order to identify distinct groups of research topics and fields. This approach allows for the revelation of the underlying research structure and trends.

**Table 4:** Top 10 highly cited articles.

**Rank**	**Article**	**Total cited**	**Year**
1	Vascular Contributions to Cognitive Impairment and Dementia A Statement for Healthcare Professionals From the American Heart Association/American Stroke Association	2,533	2011
2	National Institute of Neurological Disorders and Stroke-Canadian Stroke Network vascular cognitive impairment harmonization standards	1,162	2006
3	Prevalence, incidence, and factors associated with pre-stroke and post-stroke dementia: a systematic review and meta-analysis	1,147	2009
4	Semantic impairment in stroke aphasia vs. semantic dementia: a case-series comparison	571	2006
5	Mediterranean Diet, Stroke, Cognitive Impairment, and Depression: A Meta-Analysis	533	2013
6	Is breakdown of the blood-brain barrier responsible for lacunar stroke, leukoaraiosis, and dementia?	497	2003
7	Post-stroke dementia - a comprehensive review	370	2010
8	Association of MRI Markers of Vascular Brain Injury With Incident Stroke, Mild Cognitive Impairment, Dementia, and Mortality The Framingham Offspring Study	352	2010
9	Underestimation of Cognitive Impairment by Mini-Mental State Examination vs. the Montreal Cognitive Assessment in Patients With Transient Ischemic Attack and Stroke A Population-Based Study	344	2017
10	The Montreal Cognitive Assessment (MoCA) is superior to the Mini-Mental State Examination (MMSE) for the detection of vascular cognitive impairment after acute stroke	316	2010

### 3.7 Analysis of keywords

**Figure 9:**
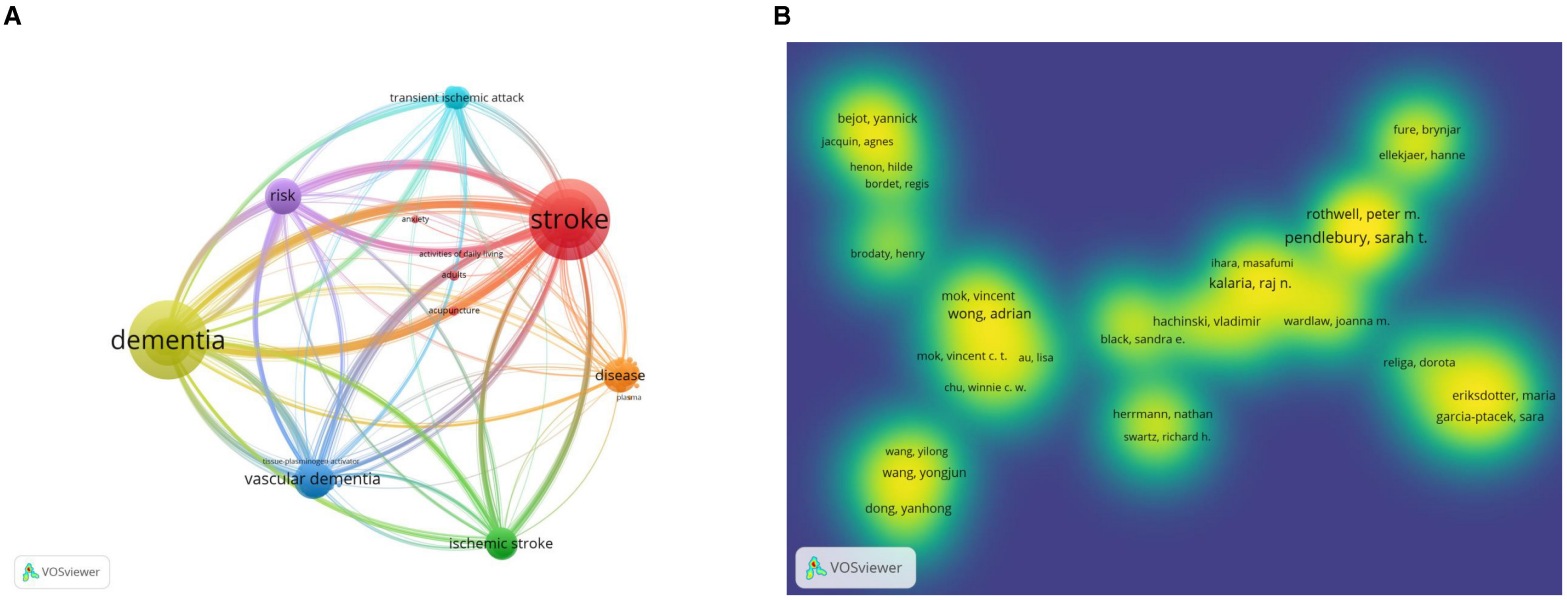
Keyword maps for research on stroke and dementia. **(A)** Visualization of the keyword network. **(B)** Cluster network of keywords.

**Figure 10:**
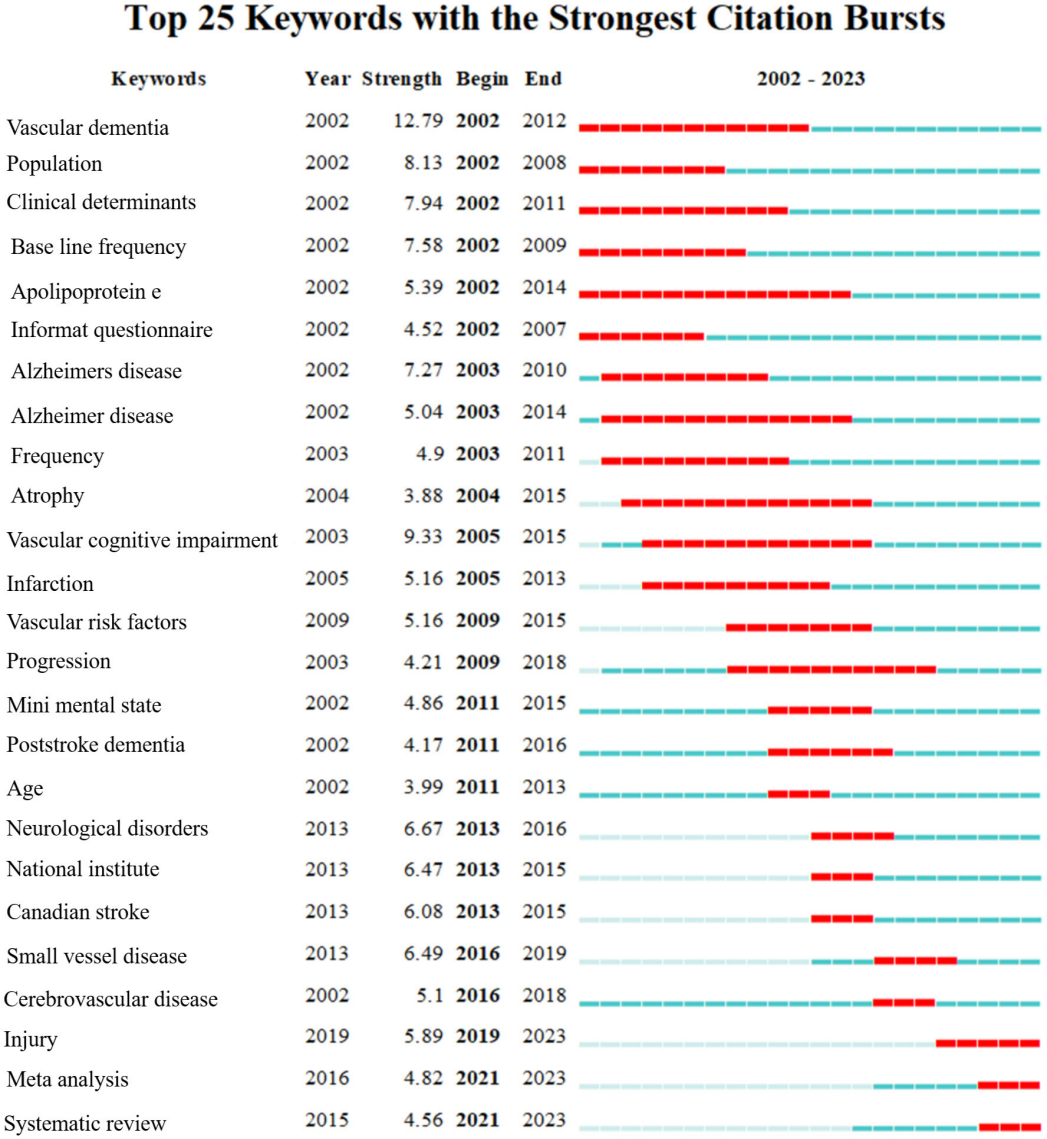
Top 25 keywords with the most cited outbreaks.

The keyword co-occurrence of 1,309 articles were analyzed in VOSviewer, by examining Figures 9A, B, it becomes clear that the prominent keywords were stroke, risk, dementia, disease, activities of daily living, and adults in relation to the topic at hand. By analyzing the keywords used in research articles, it has been noted that early studies primarily concentrated on vascular dementia, population, clinical determinants, base line frequency, apolipoprotein E, and informant questionnaire. However, over the recent two years, the research focus has shifted toward injury, meta-analysis, and systematic analysis (Figure 10). In the past, researchers only studied stroke and dementia as separate diseases in clinical studies. While the current research is focused on measuring the relationship between these two conditions. Preventing dementia by preventing stroke may be a future research trend.

## 4 Discussion

The elderly population is the fastest-growing demographic globally, with an estimated 1.5 billion individuals aged 60 and older by 2030 (31, 32). China became an aging society in 1999 (33), and its elderly population is projected to double from 10% to 20% within two decades (34). According to the World Health Organization, over 5% of the global population aged 65 and above suffers from cognitive impairment (32), with China's prevalence of mild cognitive impairment exceeding this figure at 11% (33). Alzheimer's disease is the most common cause of dementia, comprising 60–80% of cases (34, 35). Moreover, dementia risk doubles after a stroke (36), with post-stroke dementia prevalence ranging from 7% to 23% within the first year (20, 37, 38). This bibliometric analysis provides a comprehensive overview of stroke and dementia research over the past two decades. During this period, the volume of publications in this field has shown a consistent upward trend, accelerating after 2018. Both the total number of publications and citations have expanded exponentially, reflecting the growing interest in this area. This trend is likely driven by the aging population and increased disease prevalence, prompting greater attention from healthcare professionals and researchers. Advances in neuroimaging and related technologies have improved diagnostic accuracy, reducing misdiagnosis rates. Additionally, the recognition of shared mechanisms between stroke and dementia has further fueled research interest, contributing to more focused prevention and treatment efforts.

Our keyword analysis reveals evolving research trends, initially focusing on vascular dementia, clinical factors, and population characteristics. The study establishes a link between stroke-mediated vascular dementia and identifies key risk factors such as age and genetics (39–44). Recent research has shifted toward understanding the mechanisms of stroke and dementia, emphasizing post-stroke prevention and related factors. Blood-brain barrier integrity, cerebral blood flow, inflammation, and amyloid protein function have emerged as primary determinants of post-stroke dementia (45–47). Notably, atrial fibrillation has also surfaced as a significant keyword, highlighting its role as a stroke trigger that can precipitate dementia and related complications. Emerging evidence suggests that atrial fibrillation increases the risk of asymptomatic cerebral infarction, which is strongly associated with subsequent strokes and cognitive decline (41). Early stroke prevention in atrial fibrillation patients is crucial in reducing dementia risk (48). By analyzing these key terms, this study delineates research trajectories and prospective focal areas, guiding future investigations.

The analysis of countries and regions provides insights into the research status and influence of various nations in the field. It suggests that the volume of publications and the caliber of academic outputs are correlated with epidemiological indicators such as morbidity and mortality rates within these regions. Furthermore, it mirrors the variance in disease prioritization and regional policies. Among the 80 countries and territories analyzed, we found that while China leads in the number of publications, the United States exhibits a higher H-index, indicating the greater impact and importance of its research output. England's high network density and its prominent role as a collaborative hub underscore its key position in facilitating global connections among researchers and institutions. This collaborative spirit fosters the dissemination of knowledge, the exchange of ideas, and a more comprehensive understanding of the underlying mechanisms of the disease. These findings not only highlight the significance of stroke and dementia in global healthcare but also emphasize the importance of international and regional collaboration and exchange. Such collaboration will further influence the development of treatment and prevention strategies in different countries and regions (49).

The investigation into institutional contributions underscores the distinguished influence and stature of select institutions within this domain. Prominent among these are the University of Toronto, the University of Oxford, the Karolinska Institute, and Newcastle University, which have played a pioneering role in dictating the early course of research endeavors. The journal analysis brings to light the array of publication platforms dedicated to this field of study. The journal *Stroke*, distinguished by its substantial publication count, H-index, and citation frequency, affirms its role as a central hub for the propagation of influential research. This solidifies its notable impact and prestigious status within the academic community, serving as an invaluable reference for scholars. Notably, five of the top 10 highly cited articles stem from this journal, including esteemed works such as “Vascular Contributions to Cognitive Impairment and Dementia: A Statement for Healthcare Professionals from the American Heart Association/American Stroke Association” and “National Institute of Neurological Disorders and Stroke-Canadian Stroke Network Vascular Cognitive Impairment Harmonization Standards” (50, 51), offering comprehensive insights into current research trends and diagnostic criteria from distinctive perspectives.

This study provides a detailed and comprehensive analysis of the research field of stroke and dementia, exploring the current research status while identifying regional, institutional, and journal-based differences. Additionally, through keyword and reference clustering analysis, we identified key issues and trending topics in this field. First, the relationship between stroke and dementia has been well established, and further investigations into deeper risk factors such as atrial fibrillation and age are ongoing (52–54). More importantly, while preliminary insights into the underlying pathological and physiological mechanisms of stroke and dementia have been made—such as the role of vascular factors and blood-brain barrier integrity—further clarification and exploration are still required.

While this study offers a comprehensive analysis of the field of stroke and dementia, there exist several limitations. Firstly, despite our examination of publications from various regions, institutions, and journals, our study was constrained by the availability of literature and databases, potentially overlooking some pertinent research findings. Secondly, although we investigated collaborative organizations and partnerships, we did not conduct an extensive assessment of the actual impacts and outcomes of these collaborations, nor did we provide a thorough evaluation of the quality of published articles or their impact factors. Additionally, due to time constraints, some recently published high-quality articles may not fully reflect their influence given their recency. Lastly, while we have uncovered initial indications of the association and underlying pathophysiological mechanisms between stroke and dementia, additional research and empirical data are indispensable to gain a comprehensive understanding and address related issues. These limitations highlight areas for improvement, which future studies could address to achieve a more comprehensive understanding of the field.

In conclusion, this comprehensive analysis sheds light on the intricate relationship between stroke and dementia, revealing evolving research trends and pivotal areas of investigation. The findings underscore the increasing significance of this field, emphasizing the importance of collaborative efforts across nations and regions. Moreover, while highlighting current research trajectories, it underscores the need for further exploration to fully comprehend and address the complex interplay between stroke and dementia, particularly regarding their pathophysiological mechanisms and subsequent implications for treatment and prevention strategies.

## Data Availability

The raw data supporting the conclusions of this article will be made available by the authors, without undue reservation.
